# HIV/AIDS length of stay in Portugal under financial constraints: a longitudinal study for public hospitals, 2009–2014

**DOI:** 10.1186/s12913-019-4131-0

**Published:** 2019-05-10

**Authors:** Gonçalo F. Augusto, Sara S. Dias, Alexandre V. Abrantes, Maria R. O. Martins

**Affiliations:** 1 Global Health and Tropical Medicine (GHTM), Instituto de Higiene e Medicina Tropical – Universidade NOVA de Lisboa (IHMT-UNL), Rua da Junqueira 100, 1349-008 Lisbon, Portugal; 2 Epidoc Unit – CEDOC, NOVA Medical School – Universidade Nova de Lisboa (NMS-UNL), Campo Mártires da Pátria 130, 1169-056 Lisbon, Portugal; 3 Center for Innovative Care and Health Technology (ciTechCare), Escola Superior de Saúde de Leiria (ESSLei), Instituto Politécnico de Leiria (IPLeiria), Campus 2, Morro do Lena, Alto do Vieiro, Apartado 4137, 2411-901 Leiria, Portugal; 4 Health Policy and Administration Department, Escola Nacional de Saúde Pública – Universidade NOVA de Lisboa (ENSP-UNL), Avenida Padre Cruz, 1600-560 Lisbon, Portugal

**Keywords:** Austerity, HIV/AIDS, LOS, Hospitals, Portugal

## Abstract

**Background:**

The global financial crisis and the economic and financial adjustment programme (EFAP) forced the Portuguese government to adopt austerity measures, which also included the health sector. The aim of this study was to analyse factors associated with HIV/AIDS patients’ length of stay (LOS) among Portuguese hospitals, and the potential impact of the EFAP measures on hospitalizations among HIV/AIDS patients.

**Methods:**

Data used in this analysis were collected from the Portuguese database of Diagnosis Related Groups (DRG). We considered only discharges classified under MCD 24 created for patients with HIV infection. A total of 20,361 hospitalizations occurring between 2009 and 2014 in 41 public hospitals were included in the analysis. The outcome was the number of days between hospital admission and discharge dates (LOS). Hierarchical Poisson regression model with random effects was used to analyse the relation between LOS and patient, treatment and setting characteristics. To more effectively analyse the impact of the EFAP implementation on HIV/AIDS hospitalizations, yearly variables, as well as a variable measuring hospitals’ financial situation (current ratio) was included.

**Results:**

For the 5% level, having HIV/AIDS as the principal diagnosis, the number of secondary diagnoses, the number of procedures, and having tuberculosis have a positive impact in HIV/AIDS LOS; while being female, urgent admission, in-hospital mortality, *pneumocystis* pneumonia, hepatitis C, and hospital’s current ratio contribute to the decrease of LOS. Additionally, LOS between 2010 and 2014 was significantly shorter in comparison to 2009. Differences in LOS across hospitals are significant after controlling for these variables.

**Conclusion:**

Following the EFAP, a number of cost-containment measures in the health sector were implemented. Results from our analysis suggest that the implementation of these measures contributed to a significant decrease is LOS among HIV/AIDS patients in Portuguese hospitals.

## Background

The economic and financial crisis that started in 2008 reached Portugal in 2009 and had economic and social consequences that are still felt to this day. Portugal has experienced recessions in 2009 (− 2.98% in GDP), 2011 (− 1.83%), 2012 (− 4.03%) and 2013 (− 1.13%) and this was accompanied by a dramatic rise in the unemployment rate, which rose from 7.6% in 2008 to 16.2% in 2013 [1]. Due to the high level of public debt and the increasing difficulty in financing its economy, the country received a financial bailout by the European Commission, the International Monetary Fund and the European Central Bank [2].

In the Memorandum of Understanding (MoU) signed with the three institutions above, the Portuguese government compromised by implementing a number of reforms aimed at reducing public spending. With regard to the health sector, the MoU set a number of measures aimed at cost containment and increasing efficiency within the Portuguese National Health Service (NHS) [3]. These included severe cuts in the wages of health care worker; the creation and implementation of clinical guidelines; the reorganisation and rationalisation of the hospital network through specialisation and concentration of hospital and emergency services; and setting up a system for comparing hospital performance (benchmarking) [4].

The consequences of the economic and financial crisis on the health of the citizens and health care has been studied all over Europe [5–7] and has generated intense debate. However, the impact of these events on health care use is still unclear, mainly due to lack of measures to monitor the impact of the crisis and its consequences on health and health care. From the demand side, one could argue that income reductions could have an impact in the use of health care services, as international evidence shows that low-income people have a higher use of in-patient care [8], and longer in-patient stays [9], due to deterioration of their health status. From the supply side, budget cuts could have led hospitals to reduce inefficiencies but also to decrease quality of health care provided (e.g. by reducing length of stay or decreasing the number of admissions).

The impact of the crisis on the health of the population has been the focus of recent research but findings are very controversial. Following the onset of the crisis, a rise in suicides has been observed in Greece, Spain, the UK, and the USA [10–13]; and a rise in mental health disorders has been observed in Greece and Spain [14–16]. Literature also suggests that there has been an increase in cases of infectious disease, homicides, substance abuse, and poor self-reported health in Greece [10, 17–19]. In contrast, there is evidence showing that economic crisis is associated with reduced mortality related to road traffic accidents and cardiovascular events [20].

The existing evidence suggests that since austerity measures came into effect in 2011 there has been a decline in access to health care in Portugal, particularly among vulnerable population groups who do not benefit from user charges exemptions [21]. Other Southern European countries, namely Greece and Spain [22, 23], experienced a similar situation and witnessed a serious setback in terms of the universal health coverage, population well-being and welfare state as a result of austerity measures [24].

The crisis led many countries to reduce budgets earmarked for control and prevention of infectious diseases, including HIV [25, 26]. People living with HIV (PLWHIV) are vulnerable group who need constant hospital care both outpatient and inpatient) and, therefore, constitute a relevant case study to evaluate how the austerity measures imposed by the MoU had an impact in health care provision. As PLWHIV are living longer and experiencing age-associated comorbidities, hospitalizations have become an important indicator of healthcare expenditure in these patients. As in the rest of the world, in Portugal HIV-related hospitalizations are among the most expensive. In 2008, the average cost of treatment was 14,277 EUR/patient/year, with the main cost-driver being ART (EUR 9598), followed by hospitalization costs (EUR 1323) [27]. In addition, the weight of hospitalization costs was considerably higher for the most severely affected patients [27].

By identifying and characterising the variations in length of stay (LOS) among HIV/AIDS hospitalizations across different Portuguese hospitals, the aim of this paper was to analyse the potential impact of the economic and financial adjustment programme (EFAP) on HIV/AIDS patients LOS.

## Methods

### Data source

Data used in this analysis were collected from the Portuguese national database of the diagnosis related groups (AP-DRG v21.0) managed by the Central Administration of the Health System (ACSS). The DRG database is anonymous and available for scientific research. DRGs were first introduced in Portuguese hospitals through a pilot study in 1984 and, since the 1990s, DRGs are used for DRG-based hospital budget allocation from the NHS to hospitals and for DRG-based case payment from third-party payers [28].

Currently, there is only one DRG system in Portugal that applies to all NHS hospitals (public) and all patients (inpatients and ambulatory surgery), with exception of outpatients and patients treated in psychiatric and rehabilitation healthcare settings. Private hospitals are not included in the system. The DRG system currently in place defines 669 DRGs within 25 Major Diagnostic Categories (MDCs), each corresponding to one organ or physiological system [28]. The DRG system is supervised and maintained by the ACSS within the Ministry of Health.

In the DRG database, each record corresponds to a discharge episode (hospitalization) and includes information about the patient as well as information collected during the hospitalization, including age, sex, place of residence, type of admission (elective or urgent), dates of hospitalization and discharge, principal diagnosis and secondary diagnosis, procedures during hospitalization, and outcome at discharge (dead or alive).

### Study population

**Fig. 1:**
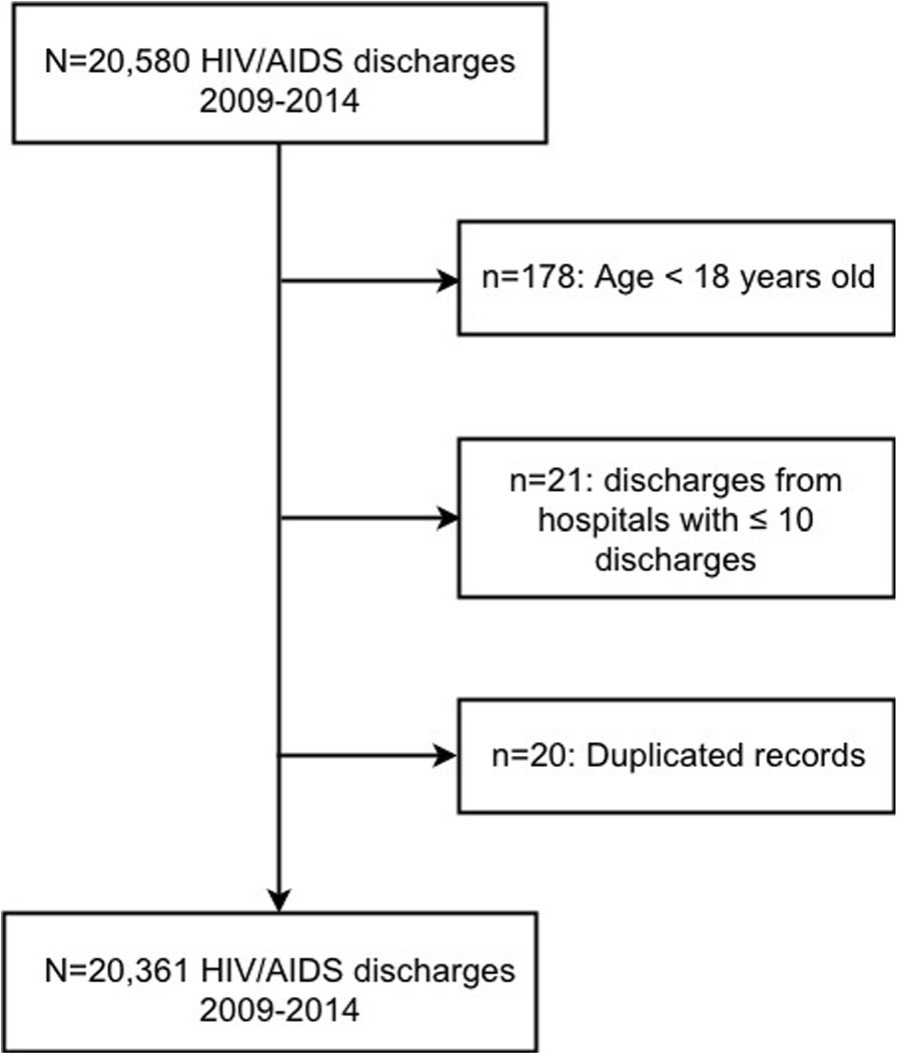
Selection profile of study population

We considered only discharges classified under MDC created for patients with HIV infection (MDC 24). Thus, the dataset provided by the ACSS included 20,580 discharges registered in public acute care hospitals in the Portuguese NHS classified under MDC 24, between 1st January 2009 and 31st December 2014. For this study we considered only those that met the following criteria: inpatients aged 18 or older; hospitalizations from hospitals with more than 10 discharges. Following these criteria, 20,361 hospitalizations occurring in 41 hospitals were included in the analysis (Fig. 1).

Unlike previous studies [29, 30], transfers were not excluded from the analysis in order to capture the dynamics of the referral system among NHS hospitals. Thus, shorter hospitalizations in smaller hospitals followed by longer hospitalizations in bigger hospitals were all included in this analysis. Length of stay was considered for each patient discharged, including patients transferred between different hospital centres (LOS was not summed), in order to capture all hospitalizations. Transfers between hospitals represented only 2.5% (*n* = 507) of the total sample.

### Outcomes and covariates

The outcome variable was the number of days between hospital admission and discharge dates (LOS). The main explanatory variable was the year, as we aimed to examine the impact of the EFAP, which was implemented in Portugal between May 2011 and May 2014.

We examined three types of covariates: patient, treatment and setting variables.

- Patient covariates considered were: gender, age (at the date of admission), region of residence, type of admission (elective or urgent), readmission within 30 days of discharge, in-hospital mortality, presence of selected co-infections (*Pneumocystis* pneumonia, Hepatitis B, Hepatitis C, and Tuberculosis), HIV/AIDS as principal diagnosis at admission, and the number of secondary diagnosis (obtained as a sum of diagnosis apart from the main diagnosis, in 19 possible cases [29, 31]);
- Treatment covariates included: number of procedures (obtained as a sum of procedures in 20 possible cases [29, 31]);
- Setting covariates included whether the hospital was merged into a Hospital Centre or not, and the hospital’s current ratio.

*Pneumocystis* pneumonia became a common manifestation of HIV infection in the developed world during the 1980s, and frequently resulted in death. Following the introduction of HAART in 1996, there was a dramatic decline in the incidence of opportunistic infections in HIV/AIDS patients (including *Pneumocystis* pneumonia). However, despite the major benefits associated with HAART, *Pneumocystis* pneumonia remains one of the most common AIDS-defining diagnoses and most common causes of AIDS-related death, especially in HIV-infected patients who present late into medical care [32]. Hepatitis B and Hepatitis C are also common co-infections among people living with HIV. The estimated prevalence of hepatitis B among people living with HIV is 5–20%; thus, approximately 2 to 4 million people living with HIV worldwide have chronic hepatitis B coinfection [33, 34]. It is estimated that hepatitis C affects 2–15% of people living with HIV worldwide (and up to 90% of those are people who inject drugs [35]. Likewise, Tuberculosis and HIV/AIDS constitute the main burden of infectious disease in resource-limited countries [36]. Some 14 million individuals worldwide are estimated to be dually infected with HIV and Tuberculosis [37] and TB remains the leading cause of death among people living with HIV [38]. The DGR database records the principal and all secondary diagnosis (up to 19) from each discharge using ICD-9 codes. Table 1 shows the ICD-9 codes used to identify HIV and selected co-infections in the DRG dataset provided.

**Table 1:** ICD-9 codes and description for the selected diagnosis

ICD-9 codes	Description
070.2–070.3	Hepatitis B
070.7	Hepatitis C
042	Human immunodeficiency virus (HIV) infection
136.3	Pneumocystosis
010–018	Tuberculosis

In the beginning of the 2000s, the NHS hospital network was reformed. Firstly, hospitals were transformed in public enterprises (2005) with the aim of promoting autonomous management and improve efficiency. Secondly, some hospitals were grouped into Hospital Centres. The rationale behind the creation of Hospital Centres was to improve efficiency through better coordination between institutions providing hospital care in the same geographical area [39]. The process of merging hospitals toke place over for several years, this explains why there were important changes during the study period (2009–2014): in 2009 there were 46 hospital institutions and in 2014 there were 41, and therefore different codes in the dataset provided by ACSS correspond currently to the same Hospital Centre. In order to have the same number of institutions during the study period, hospitals were coded according to their current status, as to simulate the Hospital Centre of which they are currently part of, and a dummy variable was added to measure the effect of this merger.

Finally, a variable measuring the hospital’s financial situation was added to this analysis. The current ratio is a liquidity ratio that measures a company’s ability to pay short-term and long-term obligations. To measure this ability, the current ratio considers the current total assets of a company (both liquid and illiquid) relative to that company’s current total liabilities [40], as follows:

$$ Current\ Ratio=\frac{Current\ Assets}{Current\ Liabilities} $$

The annual current ratio for each hospital institution in the DRG dataset was taken from the annual report and accounts from each hospital between 2009 and 2014.

### Statistical analysis

The skewness and heterogeneity of LOS is a challenge for statistical analysis [41, 42]. Particularly, HIV/AIDS LOS has 6–7% of outliers and its distribution is very asymmetric [31]. LOS has been analysed using many different methods. For example, Barbour et al. studied changes among HIV/AIDS inpatients using a multivariable linear regression model [43], while Huang et al. analysed LOS and costs based on a generalized linear mixed model [44]. Other authors, like Wang et al., analysed maternity LOS from a two-component Poisson mixed model [45]. However, researchers must take into consideration that hospitalizations from the same hospital are often correlated, since ignoring the dependence of clustered data may lead to illegitimate associations and false interpretations [42].

Hierarchical Poisson regression model was specified to analyse the relation between LOS and the covariates. In DRG data, patients are nested within hospitals on the basis of their own choices which can range from place of residence, trust in a particular doctor, or even the hospital’s reputation. This important element breaks the independence assumptions of classical regression analysis. Hence, hierarchical modelling is considered a more suitable statistical method when using multilevel structured data, like patients clustered within hospitals [46]. Additionally, the recognition of hospital random effects, which are nevertheless important, can be used to explain variations in hospital quality/performance [42].

Let y_ij_ (i = 1, 2, …m; j = 1, 2, …n_i_) the count variable (LOS) of the j ^th^ observation (hospitalizations) in the i ^th^ hospital, where m is the number of hospitals and

$$ {\sum}_{\mathrm{i}=1}^{\mathrm{m}}{\mathrm{n}}_{\mathrm{i}}=\mathrm{n} $$

is the sample size. The generalized linear model takes the form:

$$ {\uptheta}_{\mathrm{i}\mathrm{j}}={\upeta}_{\mathrm{i}\mathrm{j}}={\upchi}_{\mathrm{i}\mathrm{j}}\upbeta +{\upnu}_{\mathrm{i}} $$

where χ_ij_ is vector with covariates with regression coefficients β, and ν_i_ is assumed to be independent and normal distribution.

We used a mediation analysis to check if year dummies vary whether the current ratio is included or not.

All statistical analyses were performed using statistical software R and its library MASS and package *glmmPQL*.

## Results

The overall median length of stay (LOS) was 11 days (IQR = 16). Table 2 shows the summary statistics of hospitalization according to discharge episodes characteristics.

**Table 2:** Characteristics of HIV discharges in Portuguese NHS hospitals, 2009–2014

Variables	2009	2010	2011	2012	2013	2014	Total
Discharges, N	3864	3735	3576	3538	3146	2502	20,361
Length of stay (days); median (IQR)	11 (17)	11 (16)	11 (16)	11 (16)	11 (16)	11 (16)	11 (16)
Age (years); median (IQR)	41 (14)	43 (15)	44 (15)	44 (16)	45 (15)	46 (15)	44 (15)
No. secondary diagnoses; median (IQR)	6 (4)	6 (5)	6 (5)	7 (6)	8 (6)	8 (6)	7 (5)
No. procedures; median (IQR)	8 (6)	8 (6)	8 (6)	8 (6)	8 (7)	9 (7)	8 (7)
Gender; n (%)							
Male	2783 (72.02)	2658 (71.16)	2571 (71.90)	2538 (71.74)	2282 (72.54)	1796 (71.78)	14,628 (71.84)
Female	1081 (27.98)	1077 (28.84)	1005 (28.10)	1000 (28.26)	864 (27.46)	706 (28.22)	5733 (28.16)
Region of residence; n (%)							
North	1025 (16.53)	93 (25.11)	860 (24.05)	856 (24.19)	803 (25.52)	568 (22.70)	5050 (24.80)
Centre	472 (12.22)	526 (14.08)	488 (13.65)	466 (13.17)	436 (13.86)	368 (14.71)	2756 (13.54)
Lisbon and the Tagus Valley	1986 (51.40)	1968 (52.69)	1950 (54.53)	1920 (54.27)	1628 (51.75)	1396 (55.80)	10,848 (53.28)
Alentejo	50 (1.29)	45 (1.20)	39 (1.09)	40 (1.13)	34 (1.08)	29 (1.16)	237 (1.16)
Algarve	258 (6.68)	203 (5.44)	213 (5.96)	232 (6.56)	227 (7.22)	125 (5.00)	1258 (6.18)
Other	73 (1.89)	55 (1.47)	26 (0.73)	24 (0.68)	18 (0.57)	16 (0.64)	212 (1.04)
Type of admission; n (%)							
Elective	533 (13.79)	14.40)	590 (16.50)	726 (20.52)	567 (18.02)	426 (17.03)	3380 (16.60)
Urgent	3331 (86.21)	3197 (85.60)	2986 (83.50)	2812 (79.48)	2579 (81.98)	2076 (82.97)	16,981 (83.40)
Readmission within 30 days; n (%)							
No	3405 (88.12)	3263 (81.36)	3177 (88.84)	3141 (88.78)	2817 (89.54)	2227 (89.01)	18,030 (88.55)
Yes	459 (11.88)	472 (12.64)	399 (11.16)	397 (11.22)	329 (10.46)	275 (10.99)	2331 (11.45)
In-hospital mortality; n (%)							
No	3306 (85.56)	3248 (86.96)	3140 (87.81)	3131 (88.50)	2766 (87.92)	2204 (88.09)	17,795 (87.40)
Yes	508 (14.44)	487 (13.04)	436 (12.19)	407 (11.50)	380 (12.08)	298 (11.91)	2566 (12.60)
HIV/AIDS as principal diagnosis; n (%)							
No	953 (24.66)	979 (26.21)	1075 (30.06)	998 (28.21)	1108 (35.22)	512 (20.46)	5625 (27.63)
Yes	2911 (75.34)	2756 (73.79)	2501 (69.94)	2540 (71.79)	2038 (64.78)	1990 (79.54)	14,736 (72.37)
*Pneumocystis* pneumonia; n (%)							
No	3610 (93.43)	3439 (92.07)	3315 (92.70)	3284 (92.82)	2920 (92.82)	2300 (91.93)	18,868 (92.67)
Yes	254 (6.57)	296 (7.93)	261 (7.30)	254 (7.18)	226 (7.18)	202 (8.07)	1493 (7.33)
Hepatitis B; n (%)							
No	3699 (95.73)	3580 (95.85)	3382 (94.57)	3386 (95.70)	2985 (94.88)	2379 (95.08)	19,411 (95.33)
Yes	165 (4.27)	155 (4.15)	194 (5.43)	152 (4.30)	161 (5.12)	123 (4.92)	950 (4.67)
Hepatitis C; n (%)							
No	2796 (72.36)	2726 (72.99)	2530 (70.75)	2520 (71.23)	2283 (72.57)	1802 (71.02)	14,657 (71.99)
Yes	1068 (27.64)	1009 (27.01)	1046 (29.25)	1018 (28.77)	863 (27.43)	700 (27.98)	5704 (28.01)
Tuberculosis; n (%)							
No	2118 (54.81)	2039 (54.59)	2008 (46.15)	2100 (59.36)	1828 (58.11)	1382 (55.24)	11,475 (56.36)
Yes	1746 (45.19)	1696 (45.41)	1568 (43.85)	1438 (40.64)	1318 (41.89)	1120 (44.76)	8886 (43.64)

Out of 20,361 discharges, 14,628 (71.8%) were male and the median age was 44 years (IQR = 15). During the study period, the median number of secondary diagnoses was 7 (IQR = 5) and the median number of procedures was 8 (IQR = 7). The majority of hospitalizations corresponded to patients living in the Lisbon and the Tagus Valley region (53.3%). During the study period (2009–2014), there was a steady decrease in the number of hospitalizations (Table 2), while the majority corresponded to urgent admissions (83.4%). The most common HIV-related infections among hospitalizations between 2009 and 2014 were tuberculosis (43.6%) and hepatitis C (28.0%). In-hospital mortality during the same period was 12.6%.

The hierarchical Poisson model as estimated by penalized quasi-likelihood and the majority of covariates have a significant impact in LOS (Table 3). Although age is not statistically significant, it was retained in the model to control for possible confounding.

**Table 3:** Hierarchical Poisson regression models estimation for HIV/AIDS LOS, 2009–2014

Variables	Model 1		Model 2	
	Coefficient	*p*-value	Coefficient	*p*-value
Intercept	1.668	< 0.001	1.837	< 0.001
Gender (Female)	−0.041	0.001	−0.039	0.011
Age	−0.001	0.118	−0.001	0.137
Year 2010 (reference 2009)	−0.060	0.006	−0.092	< 0.001
Year 2011 (reference 2009)	−0.068	0.003	− 0.109	< 0.001
Year 2012 (reference 2009)	−0.135	< 0.001	−0.186	< 0.001
Year 2013 (reference 2009)	−0.214	< 0.001	−0.268	< 0.001
Year 2014 (reference 2009)	−0.238	< 0.001	−0.262	< 0.001
No. secondary diagnoses	0.044	< 0.001	0.043	< 0.001
No. procedures	0.084	< 0.001	0.085	< 0.001
Urgent admission	−0.069	< 0.001	− 0.068	< 0.001
Readmission within 30 days	− 0.039	0.079	− 0.040	0.080
In-hospital mortality	−0.139	< 0.001	−0.142	< 0.001
HIV/AIDS as principal diagnosis	0.084	< 0.001	0.085	< 0.001
Pneumocystis pneumonia	−0.131	< 0.001	−0.129	< 0.001
Hepatitis C	−0.126	< 0.001	−0.126	< 0.001
Tuberculosis	0.392	< 0.001	0.391	< 0.001
Hospital merge	0.081	0.001	0.046	0.066
Current ratio	–	–	− 0.144	< 0.001

In contrast with the estimated coefficient of the variable that measured hospital mergers, estimated coefficients of year dummies remained statistically significant after introducing the variable current status in the model (Table 3). Thus, adjusting for other factors, patients hospitalized during 2010 and 2011 had an estimated LOS 0.092 and 0.109% lower, respectively, than those hospitalized in 2009, while patients hospitalized in 2012, 2013 and 2014 had an estimated LOS 0.186, 0.268 and 0.262% lower than those hospitalized in 2009 (Table 3).

Adjusting for other variables, estimated LOS was lower for hospitalizations resulting in death, for women, and for patients with urgent admission (Table 3). Patients with urgent admission had an estimated LOS 0.068% lower than those with elective admission (Table 3). In contrast, patients with higher number of diagnosis (or higher number of procedures) have a higher estimated HIV/AIDS LOS. Adjusting for other variables, one additional number of secondary diagnosis increased LOS by 0.043%, while one additional number of procedures increased LOS by 0.085% (Table 3).

Adjusting for other factors, when analysing selected co-morbidities, patients co-infected with *Pneumocystis* pneumonia and hepatitis C had an estimated LOS 0.129 and 0.126% shorter, respectively, than those without those co-infections (Table 3). In contrast, patients co-infected with tuberculosis had an estimated LOS 0.391% longer than those without TB. Finally, patients having HIV/AIDS as a principal diagnosis had an estimated LOS 0.085% longer than those with other principal diagnosis (Table 3).

**Fig. 2:**
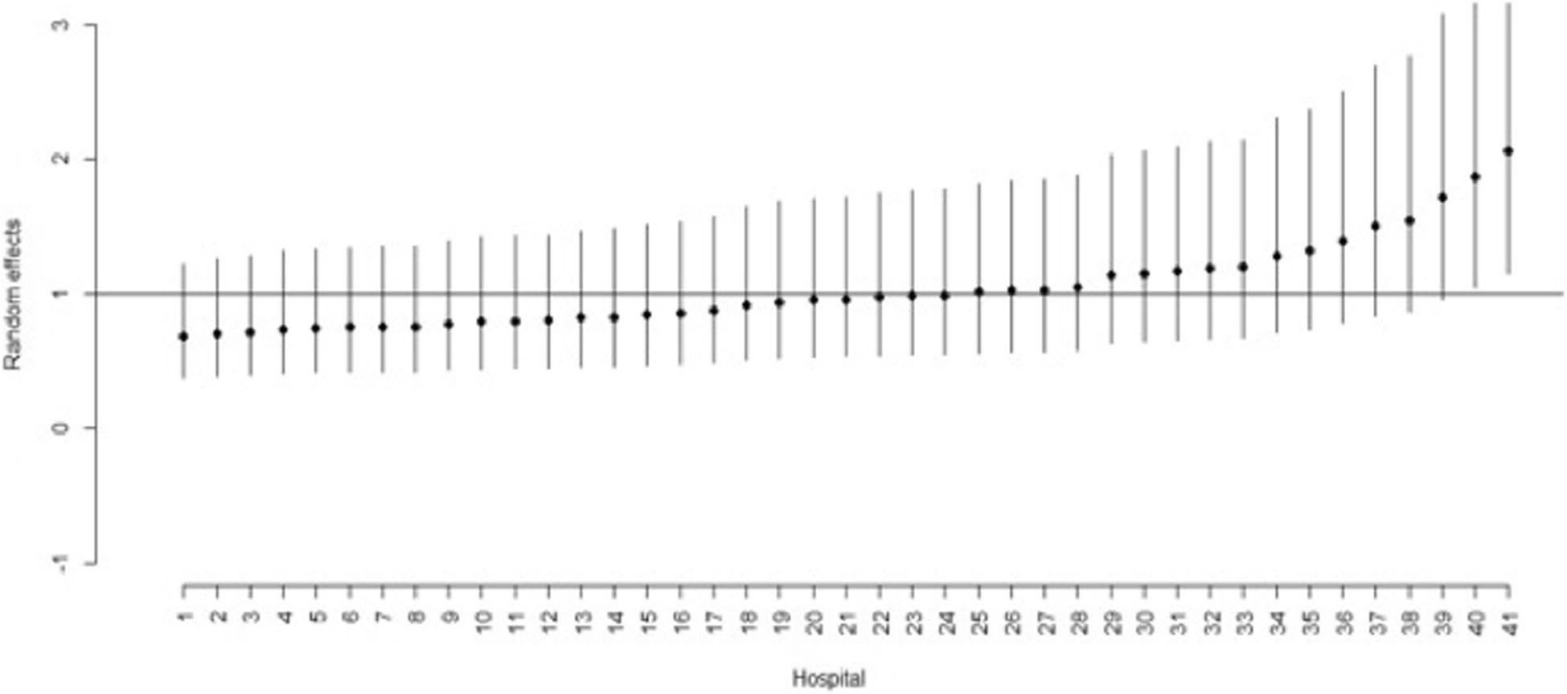
Random effects and 95% CI for each hospital

Hospital random effects were estimated to capture differences in unexplained variance in LOS across hospitals, after controlling for all other characteristics. Figure 2 shows these random effects and their respective 95% confidence intervals (IC) for the 41 hospitals analysed.

For the period 2009–2014, most hospitals had an estimated random effect closer to the mean value (one). However, two hospitals (40 and 41) showed a large positive effect, extending patients’ length of stay.

## Discussion

The constant decline in HIV-related hospitalizations during the period 2009–2014 is in line with what was observed in other studies [47]. In fact, the decrease in HIV incidence observed in Portugal suggests success in controlling the HIV epidemics in Portugal, following the worldwide trend [48]. In our analysis, most HIV patients (71.8%) hospitalized during the study period were men, which can be explained by the fact that, like in the rest of Europe, most of HIV patients in Portugal are men [49]. Therefore, estimated LOS was lower for female patients and longer for male patients. Over the study period, the median age of HIV patients hospitalized increased slightly, suggesting that PLWHIV are living longer, as demonstrated by other studies [47]. While urgent admissions have decreased steadily between 2009 and 2014 (− 37.7%) – having dropped by 10.4% between 2009 and 2011 and by 26.2% between 2012 and 2014 – elective admissions increased until 2012 (+ 36.2%) but declined in the following 2 years (− 41.3%).

This study specifies a hierarchical Poisson regression to model HIV/AIDS LOS in Portuguese public hospitals. The estimated LOS of HIV/AIDS patients hospitalized in each year between 2010 and 2014 was significantly shorter than those hospitalized in 2009. A recent study carried out in Portugal analysed all in-patient stays at all Portuguese NHS hospitals over the 2001–2012 period and found that the volume of in-patient stays, particularly non-elective stays, increased significantly, while the length of stay has become shorter and elective admissions have decreased [1]. Although our analysis included HIV/AIDS patients only, and the study period was different, we found similar results regarding the shorter LOS and the decrease of elective admissions.

The decrease of LOS for HIV/AIDS hospitalizations found in our analysis could be explained by two different hypotheses. The first one is that the EFAP measures might have induced efficiency gains, improving response from healthcare units. In contrast, the EFAP measures might have reduced quality of care provided in hospitals, with a reduction of the number of in-patient beds and increasing pressure to reduce LOS and cut costs [50]. However, our findings are not sufficient to support one hypothesis over another and further research is needed. Our results showed that the hospital’s financial situation affected HIV/AIDS patients’ hospitalizations: a greater current ratio decreased estimated LOS. This finding is supported by other studies that show a strong negative association between LOS and hospitals’ operating margins [51]. Long hospitalizations consume many hospital resources and are, therefore, associated with increasing costs. The year dummies remained statistically significant in Model 2, even after introducing the variable measuring hospitals’ current status. The fact that the annual decrease in LOS for HIV/AIDS patients was not explained by the hospitals’ current status, suggests a generalized pressure to reduce costs not fully related with the hospitals’ financial situation. By 2011, NHS hospitals were facing a severe financial situation, with the total amount of arrears (accounts payable to domestic suppliers past due date by 90 days) reaching EUR 3.0 billion [2]. Following the economic and financial adjustment programme (EFAP), a number of cost-containment measures and actions aimed at increasing efficiency in the health sector were implemented between 2012 and 2014. The Memorandum of Understanding clearly established the reduction of hospitals’ operating costs as a priority, which is the reason why NHS hospitals were under continuous pressure to cut costs during the period of the EFAP. Our findings suggest that this was an important contributor for the decrease of LOS among HIV/AIDS patients.

While patients’ age is not statistically significant at the 5% level, when adjusting for other factors the estimated LOS was significantly lower for patients who died, suggesting that mortality occurs mostly at an early stage of hospitalization. This means that there is high mortality among those patients who are admitted at the hospital in more severe stages of AIDS-related illness, as supported by other studies [42]. Both the number of secondary diagnoses and the number of procedures significantly increase LOS, suggesting longer hospitalizations. A greater number of diagnoses or procedures suggests a more severe condition of the patient admitted and therefore leads to a delayed discharge [52]. Also, estimated LOS was longer for patients who had HIV/AIDS as principal diagnosis, suggesting that those patients are admitted in a more severe condition and are therefore more likely to need a longer hospitalization.

The estimated LOS for HIV/AIDS patients was shorter for urgent admissions. It is important to highlight that, in Portugal, urgent admissions do not necessarily reflect emergency situations, as noted by previous studies [42]. Due to difficulties in accessing lower levels of care, it is not uncommon that patients seek assistance directly at a hospital emergency service, thus bypassing primary healthcare [53].

The variable measuring the effect of hospital mergers into hospital centres on estimated LOS for HIV/AIDS patients was not statistically significant in Model 2, after introducing the variable current status. Mergers can be a way of eliminating excess capacity and cutting costs, and additionally they can address performance issues for particular units or services. Hospital mergers in Portugal began in 1999 but were intensified in recent years, as a result of the economic and financial adjustment programme (EFAP) [4]. By concentrating within the same administration hospitals operating in the same geographic area and offering the same services, the aim was to increase efficiency and promote economies of scale. However, results from our study suggest the opposite, considering LOS as an indicator of hospital efficiency. Regarding recent mergers, the literature suggests that there are economies of scale and scope to explore further, but only mergers of relatively small and similar hospitals have been successful [54]. In fact, hospital mergers in Portugal did not achieve the expected efficiency gains due to the heterogeneity and geographical dispersion of many hospitals. As a result, despite being under the same administration, many hospitals kept the same practices as they had prior to being merged with other hospitals.

Estimated hospital random effects suggest differences amongst hospitals which also require the need for further research. These effects which acknowledge unexplained factors that are nonetheless important, can be interpreted as differences in hospital efficiency, after controlling for all relevant factors. Hospitals 40 and 41 showed a positive effect, extending LOS for HIV/AIDS patients. Hospital 40 corresponds to a hospital centre in northern Portugal, geographically disperse and with no differentiated services, while hospital 41 is a large hospital in the Lisbon metropolitan area, offering more differentiated services.

Our study provides an analysis of relevant factors related to LOS among HIV/AIDS hospitalizations between 2009 and 2014 in Portugal. However, it is important to note that healthcare-associated infections have a high prevalence in Portugal – overall prevalence rate of 10.5% in 2013 [55] – and are responsible for greater medical costs, longer LOS, and an increase in mortality rates. Our analysis did not include other types of pneumonia or urinary tract infections, which are major complications from nosocomial infections, as covariates, but the findings for *pneumocystis* pneumonia should prompt further research.

Although the EFAP, in place between May 2011 and May 2014, and the severe economic recession in Portugal brought important social and economic consequences in Portugal, the interpretation of our findings must be carried out with caution. Between 2012 and 2014, Portugal also witnessed changes in the National Network of Long Term Care which was expanded and might have influenced the overall reduction in LOS among patients in Portuguese NHS hospitals. Our analysis did not address the potential impact of that support network. In fact, an aspect of the whole system performance that is ignored in this analysis is the impact of hospital performance on other sectors within the health system. For instance, it could be the case that the decrease of LOS is being secured at the expense of heavy workloads for rehabilitative and primary care services [56].

This study used comprehensive discharge data compiled in mainland Portugal, and these findings are more generalizable than results based on data from a single hospital. However, this study has limitations, due to the nature of the data [57]. Firstly, there are limitations regarding the retrospective collection of data for administrative purposes, which can allow for mistakes in recording information and/or variability of coding among hospitals. Secondly, the DRG database has very limited clinical information, which would have been important to better understand the clinical profile of HIV/AIDS patients (e.g. the number of years the patient is engaged in care, viral load, CD4 cell count, ART regime). To track the long-term outcomes and quality of care, further research is needed on the information system specially implemented in NHS hospitals in Portugal to capture these important components of HIV-related care (*SI.VIDA*). Also, in this analysis, the number of secondary diagnosis was used as a proxy of the number of co-morbidities, and therefore as an indicator of the patient’s condition. However, this approach reveals nothing about the severity of each secondary diagnosis and does not measure their severity. Future research could consider the use of Elixhauser Comorbidity Index or Charlson Comorbidity Index [58, 59], which have been widely utilized by health researchers to measure burden of disease and case mix.

The fact that hospital institutions were coded in the DRG database according to the hospital centre to which they belong may have prevented a more detailed analysis of the data. Although a dummy variable was considered to capture the aggregation of hospitals into hospital centres, it would be interesting to explore, within a single hospital centre, differences among institutions regarding risk-adjusted LOS.

Finally, the selected study period is also a limitation of this analysis. Although the main objective was to measure the impact of the EFAP on hospital in-patient care for PLWHIV, the analysis over a longer period would have allowed us to better identify and measure the austerity effect from the long-term trends in LOS.

## Conclusions

The subject of the impacts of the economic crisis on the health of the population has been the focus of many studies in recent years [5–7]. Health policy research in this field poses important methodological challenges as it is often difficult to distinguish austerity measures from the overall economic crisis and its impact on health systems. Therefore, the model presented in this study aims to contribute to the analysis of the effects of the economic and financial adjustment programme on a particular group of patients.

This study presents a hierarchical Poisson model to analyse LOS among HIV/AIDS patients in Portuguese public hospitals. A number of variables (HIV/AIDS as principal diagnosis, number of secondary diagnoses, number of procedures and tuberculosis) were found to increase LOS, while others (in-hospital mortality, urgent admission, *Pneumocystis* pneumonia and hepatitis C) contributed to the decrease of LOS. Our findings also show that LOS decreased during the study period, and elective admissions decreased after 2012. Our findings also showed that hospital’s current ratio was found to decrease LOS, meaning that the better the financial situation, the lower the LOS for HIV/AIDS patients. With regard to HIV/AIDS hospitalizations, two of the analysed hospitals showed a large positive effect, extending patients’ length of stay.

These findings are a contribution to the study of the effects of the austerity measures implemented in Portugal between 2011 and 2014 in hospital care provision to a particular vulnerable group of patients. Our analysis suggests that the measures in place to cut costs and increase efficiency in public hospitals contributed to the decrease of HIV/AIDS patients’ LOS.

Results from this analysis demonstrate the need to further study this issue in order to better understand the effects of the EFAP on health and health care. Additionally, it would be important to implement measures to efficiently monitor health care delivery, particularly during periods of financial constraints.

## Abbreviations

ACSS: Central Administration of the Health System [*Administração Central do Sistema de Saúde*]
ART: Antiretroviral therapy
CD4: Cluster of differentiation 4
DRG: Diagnosis Related Groups
EFAP: Economic and financial adjustment programme
HIV/AIDS: Human immunodeficiency virus/Acquired Immunodeficiency syndrome
IQR: Interquartile range
LOS: Length of stay
MDC: Major Diagnostic Category
NHS: National Health Service (Portugal)
PLWHIV: People living with HIV
